# Zebrafish Optomotor Response and Morphology Are Altered by Transient, Developmental Exposure to Bisphenol-A

**DOI:** 10.3390/jdb9020014

**Published:** 2021-04-02

**Authors:** Mikayla Crowley-Perry, Angelo J. Barberio, Jude Zeino, Erica R. Winston, Victoria P. Connaughton

**Affiliations:** 1 Department of Biology, American University, 4400 Massachusetts Ave NW, Washington, DC 20016, USA; mc1121a@student.american.edu (M.C.-P.); ab7597a@student.american.edu (A.J.B.); jz4368a@student.american.edu (J.Z.); ericawinston7@gmail.com (E.R.W.); 2 Department of Chemistry, American University, 4400 Massachusetts Ave NW, Washington, DC 20016, USA

**Keywords:** optomotor response, vision, development, endocrine disrupting compound, estrogen, BPA, Danio rerio

## Abstract

Estrogen-specific endocrine disrupting compounds (EDCs) are potent modulators of neural and visual development and common environmental contaminants. Using zebrafish, we examined the long-term impact of abnormal estrogenic signaling by testing the effects of acute, early exposure to bisphenol-A (BPA), a weak estrogen agonist, on later visually guided behaviors. Zebrafish aged 24 h postfertilization (hpf), 72 hpf, and 7 days postfertilization (dpf) were exposed to 0.001 μM or 0.1 μM BPA for 24 h, and then allowed to recover for 1 or 2 weeks. Morphology and optomotor responses (OMRs) were assessed after 1 and 2 weeks of recovery for 24 hpf and 72 hpf exposure groups; 7 dpf exposure groups were additionally assessed immediately after exposure. Increased notochord length was seen in 0.001 μM exposed larvae and decreased in 0.1 μM exposed larvae across all age groups. Positive OMR was significantly increased at 1 and 2 weeks post-exposure in larvae exposed to 0.1 μM BPA when they were 72 hpf or 7 dpf, while positive OMR was increased after 2 weeks of recovery in larvae exposed to 0.001 μM BPA at 72 hpf. A time-delayed increase in eye diameter occurred in both BPA treatment groups at 72 hpf exposure; while a transient increase occurred in 7 dpf larvae exposed to 0.1 μM BPA. Overall, short-term developmental exposure to environmentally relevant BPA levels caused concentration- and age-dependent effects on zebrafish visual anatomy and function.

## 1. Introduction

Endocrine disrupting compounds (ECDs) are exogenous chemicals of natural and synthetic origin that affect hormonal function, synthesis, and/or downstream signaling pathways [1,2]. In addition to being common aquatic contaminants [3], exposure to EDCs has been documented through contact with plastics, food and beverage packaging, pesticides, pharmaceuticals, and cosmetics [4]. Notably, EDCs are effective at low concentrations (nM to μM), levels that are consistent with the median value recorded in US streams [5,6], and mirror the modest hormonal concentrations needed to facilitate an amplified, downstream response in an organism [7]. As such, the effects of EDCs on reproductive and neuronal development have been well characterized [8,9].

While EDCs are most noted for their influence on reproductive physiology, especially on estrogens and estrogenic pathways, developmentally irregular estrogen synthesis is associated with thinning [10] and apoptosis in retina [11], corneal thinning [12], and abnormal and delayed eye growth [13]. Age-related estrogen level changes in humans are associated with neurodegenerative retinal diseases [14] and estrogen modulation as a clinical treatment for breast cancer has been linked to retinal hemorrhaging [15], macular edema [16], and color perception changes [15], underscoring the role of estrogen-specific EDCs in more than reproductive development. Yet, despite the viable connection between estrogen and vision, we still know very little about the long-term impact of altered estrogenic signaling on visual sensation, perception, and system development.

Zebrafish are a well-established vertebrate model for vision, development, and molecular biology [17]. Logistically, zebrafish are easily maintained, low cost and available year-round. Rapid and prolific asynchronous breeders, their embryos develop externally and are easily accessible, allowing for non-invasive exposure to experimental conditions and direct observation of development [18,19]. Zebrafish central nervous and endocrine system development are similar to that of humans [20,21]; zebrafish and humans also possess similar retinal anatomy [22]. Zebrafish retinal anatomy, physiology, and visually guided behaviors are well known [23,24,25], and the role of estrogen signaling pathways in both developmental and adult neurogenesis are well established [10,26,27]. Moreover, altered visually guided behaviors have been correlated with altered retinal physiology [28,29,30].

Bisphenol-A (BPA), also known as 2,2-bis(4-hydroxyphenyl)propane, is a weak estradiol agonist and known aquatic contaminant predominantly used in the manufacture of polycarbonate and epoxy resins. BPA is present in plastic water bottles, food containers, and dental sealants [5] resulting in significant levels in ~93% of the population [31], BPA is effective at extremely low doses, can be measured in various human tissues and fluids [32,33,34], and is able to cross the placenta [34,35,36], resulting in comparable titers in human fetuses [34,37]. Though the 2004 National Health and Nutrition Examination Survey found BPA present in 96% of the pregnant women studied [38], at present there is no published work on the developmental effects of BPA on humans. However, it has been shown that in utero exposure to BPA causes multiple behavioral deficits in rodents [9,39,40,41] and there is a growing body of BPA-related zebrafish work.

In fish, the effects of BPA exposure on estrogen signaling are similar to those of exogenous estradiol [42,43]. BPA binds estrogen receptors [44] in the brain, and increases the expression of estrogen receptor mRNA [45]. In zebrafish, larvae embryonically exposed to BPA are hyperactive [46,47], likely due to BPA’s deleterious impacts on neurogenesis, including decreased estrogen-induced spine formation in the prefrontal cortex and hippocampus [35], reduced survival of nascent hippocampal neurons [40], poor outgrown of motor neurons [48], and reduced midbrain size [49]. Sensory systems are also impacted: zebrafish developmentally exposed to BPA possess malformed otoliths [50] and display inhibited regeneration of lateral line neuromasts [51], the second continuing even after treatment with BPA has stopped [52]. Clearly, a functional estrogen signaling system is critical for sensory development, and early developmental exposure to BPA has long-term, adverse effects. However, the long-term consequences of developmental estrogenic manipulation on vision remain undefined.

We hypothesized that transient developmental exposure to environmentally relevant concentrations of BPA will have long-term effects on the visual system. We assessed visual system function using the optomotor response (OMR), a reliable, vision-based behavior; changes in length and eye size were also assessed. Our results show BPA exposure increases the number of fish displaying a positive OMR, an effect observed 1 and 2 weeks after removal from treatment. These behavioral differences depend on developmental age, with the largest differences occurring when exposure occurred at 72 hpf and 7 dpf, when significant differences in eye diameter were also noted.

## 2. Materials and Methods 

### 2.1. Animal Maintenance

All experiments were approved by the Institutional Animal Care and Use Committee (IACUC) of American University (Protocols #1700 and #20-03). Wild type zebrafish (*Danio rerio*) embryos and larvae were obtained from in-house spawning at the American University Fish Facility, or from a commercial supplier (LiveAquaria, Rhinelander, WI, USA). Adult zebrafish were maintained in an Aquatic Habitat (AHAB, Pentair, Apopka, FL, USA) system at 28–29 °C, on a 14-h light: 10-h dark photoperiod and were fed Tetramin and live brine shrimp twice daily [53,54]. To obtain eggs, 5–6 adult females and 3–4 adult males were placed in a 16” mesh-bottomed breeding chamber overnight. The following morning, approximately one hour after light onset, fertilized eggs were collected, staged (shield to 75% epiboly), cleaned, and maintained at the same photoperiod and water temperature as adults until placed in experimental treatment conditions. Embryos and larvae were housed in 100 mm glass Petri dishes in temperature-controlled incubators (Heratherm, ThermoFisher, Waltham, MA, USA), at densities of ≤ 50. Embryos and larvae were transferred to new dishes of system water daily, during which time larval mortality was also recorded; larvae were fed AP100 (Aquatic Habitats/Pentair, Apopka, FL, USA) once daily and were transferred to new dishes of system water after 30 min of feeding. Embryos received from the commercial supplier were cleaned and staged upon arrival in the lab and maintained as described above.

### 2.2. Exposures

At 24 h post-fertilization (24 hpf), 72 h post-fertilization (72 hpf) or 7 days post-fertilization (7 dpf), embryos or larvae were placed into one of four experimental treatments for 24 h: (1) system water control, (2) dimethyl sulfoxide vehicle control (DMSO, Sigma Chemical Co., St. Louis, MO, USA), (3) low (0.001 μM), or (4) high (0.1 μM) BPA (TCI, Portland, OR, USA). The final concentration of DMSO was 0.0003% in the high BPA exposure group and 0.000003% in the low BPA exposure group. The higher DMSO concentration (0.0003%) was used for the vehicle control. These exposure ages were selected based on their correspondence with key events in zebrafish visual system development, specifically: development of the neural retina at 24 hpf [24], the vertical transduction pathway becomes functional at 72 hpf [24], and at 7 dpf OMRs are reliably evoked [55], as well as the presence of active estrogen synthesis (aromatase activity) in the brain [56]. BPA concentrations were selected based on their use in other studies [46,47,48,52,57,58,59] and because they are comparable to reported levels in natural water systems [6] and human tissue [32,33]. After 24 h of exposure, embryos/larvae were removed from treatment, returned to new dishes of system water, and allowed to recover for 1 or 2 weeks.

### 2.3. Behavioral Assays

Embryonic (24 hpf) and early larval (72 hpf) exposure groups were assessed for startle response and OMR changes after 1 and 2 weeks of recovery. The older exposure group, 7 dpf, was assessed immediately after exposure and after 1 and 2 weeks of recovery. These time points were selected because OMRs are not reliably elicited before 7 dpf [55,60,61]. All behavioral assays occurred in system water at 28–29 °C and were recorded with a Canon FS40 handheld video camera that was mounted directly above the behavioral chamber. Recordings were analyzed by a trained, blinded observer. 

#### 2.3.1. Startle Response

The startle response was assessed following established protocols [62,63,64]. Briefly, ≤ 10 larvae were placed in a 100 mm Petri dish and allowed to acclimate for 30 s. The dish was located inside a black-lined behavioral chamber to prevent external stimuli from confounding the results. A handheld video camera (Canon, Melville, NY, USA) was mounted above the dish so larval responses could be recorded. After acclimation, the startle stimulus (a 200 g weight) was dropped from a uniform height adjacent to the chamber. Larval responses recorded for 60 s and fish were assessed as to whether they responded to the stimulus or not. Fish that did not display a startle response or otherwise did not appear to move were removed from further analysis.

#### 2.3.2. Optomotor Response

Optomotor responses (OMRs) were recorded following published protocols [65,66]. In brief, OMRs were elicited using a Fourier motion stimulus, displayed as a rotating black and white pinwheel projected onto a 36 inch Dell flat screen computer monitor directly below the fish. The stimulus was generated using PsychoPy software (PsychoPy^3^; Nottingham, United Kingdom) and a MacBook Pro laptop (Apple, Cupertino, CA, USA) [65].

To evoke the OMR, larvae (≤ 10 at a time) were placed into a 100 mm Petri dish, the sides of which were covered with white tape to reduce external distractions. The Petri dish was placed on top of the monitor and the fish were allowed to acclimate for 1 min. A small cylindrical object was placed into the center of the chamber to provide an annulus where the fish could swim and to cover the convergence point of the pinwheel. The pinwheel rotated clockwise for 30 s and then counterclockwise for 30 s with a gray resting screen in between. This stimulus sequence was presented twice to each group of fish in succession. Larvae were considered to have a positive OMR when they swam in the direction of the stimulus and such that their heads were oriented at a 55–105 angle in respect to the midline of the stimulus. Scan sampling of recorded videos was used to identify fish with a positive OMR [66].

### 2.4. Morphometric Analysis

After behavioral assessments, larvae were anesthetized and euthanized in a 0.02% tricaine methanesulfonate solution, flash frozen, and stored at −80 °C. All sampled larvae were flash frozen to increase potential use in subsequent assays. Those that were to be measured (4–5 per age and treatment group) were thawed and placed in 4% paraformaldehyde. Fixed larvae were then imaged using a Leica MZ10F stereomicroscope fitted with a DFC700T digital camera (Leica Microsystems, Heerbrugg, Switzerland) and pictures of individual larvae were captured with accompanying software (LAS v. 4.6.1). Notochord length and eye diameter (anterior-posterior) were measured from captured images using ImageJ (https://imagej.nih.gov/ij/download.html, accessed on 11 February 2021). Each measurement was made three times and then averaged to reduce error.

### 2.5. Statistical Analysis

Survival was analyzed using a general linearized model, with treatment as the fixed factor and day as the co-variate. Survival of larvae exposed at 7 dpf was analyzed by one-way ANOVA, followed by a Tukey post hoc test at the 1- and 2 week recovery points. Differences in notochord length and eye diameter across treatment were also assessed using a one-way ANOVA followed by a Tukey post hoc test at each exposure age. Eye diameter measurements were normalized to notochord lengths prior to statistical analysis. Startle responses were analyzed using a one-way ANOVA performed separately for each age and treatment group. If significance was noted, a Tukey post hoc test was performed to identify differences. OMR was analyzed using a one-way ANOVA, followed by a post hoc multiple comparison (with a Bonferroni correction). All statistical tests were performed with SPSS software (v. 27, IBM, Armonk, NY, USA) and evaluated at an α-level of 0.05.

## 3. Results

### 3.1. Suvival

**Figure 1:**
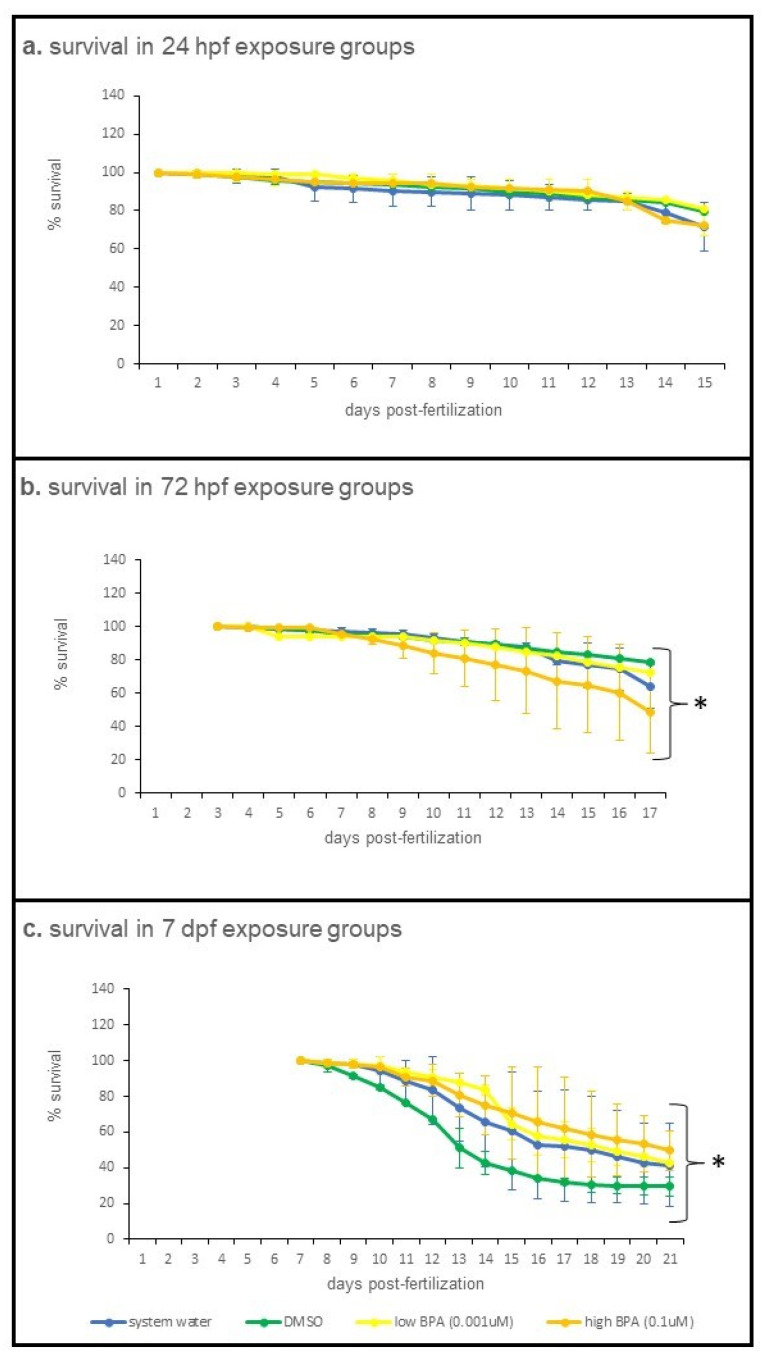
Survival trends across all exposure ages and recovery sampling points. Survival was analyzed using a general linearized model, with treatment as the fixed factor and day as the co-variate. Values are reported as mean percent daily survival ± SD for each treatment group. (**a**) Overall daily survival of larvae exposed at 24 hpf was unaffected by treatment (*p* = 0.238). For 24 hpf exposures, initial populations were as follows: system water = 341; DMSO = 250; low BPA = 355; high BPA = 250. (**b**) Overall daily survival of larvae exposed at 72 hpf was significantly impacted by treatment (*p* < 0.001). For 72 hpf exposures, initial populations were system water = 293; DMSO = 250; low BPA = 298; high BPA = 250. (**c**) Overall daily survival of larvae exposed at 7 dpf was significantly impacted by treatment (*p* = 0.005). For the 7 dpf exposure groups, survival between treatment groups was assessed at 1 week and 2 week postexposure using one-way ANOVA, followed by a Tukey post hoc test; survival at these points was not significantly different: *p* = 0.191 at 1 week (14 dpf), and *p* = 0.811 at 2 weeks (21 dpf). For 7 dpf exposures, initial populations were system water = 231; DMSO = 221; low BPA = 293; high BPA = 226. Asterisks denote significant differences.

Daily survival was compared across treatment groups for each exposure age (Figure 1). Treatment was not found to significantly affect survival of larvae exposed when they were 24 hpf (*p* = 0.702; Figure 1a) with ~80% of larvae surviving across all treatment groups. In contrast, differences across day were significant (*p* < 0.001), reflecting the lower survival at the end of the experiment. There was no significant treatment * day interaction (*p* = 0.238), however, further supporting no differences in survival across treatment groups.

For larvae exposed at 72 hpf, both treatment (*p* = 0.012; Figure 1b) and day (*p* < 0.001) significantly impacted survival, and there was a significant treatment * day interaction (*p* < 0.001). Survival across all treatments was constant for the first 5–6 days of recovery, after which time survival in the low BPA treatment group decreased compared to the other treatment groups, resulting in ~50% survival at the end of the experiment. 

Treatment did not significantly impact survival of larvae exposed at 7 dpf (*p* = 0.598; Figure 1c), though day was identified as a significant main effect (*p* < 0.001). There was also a significant treatment * day interaction (*p* = 0.005), with the lowest survival overall observed in the vehicle control (DMSO) group. However, subsequent analysis at the 1 week and 2 week post-exposure time points identified no significant differences (*p* = 0.191 and *p* = 0.811, respectively) across exposure groups.

### 3.2. Morphometric Results

#### 3.2.1. 24 hpf Exposures

**Figure 2:**
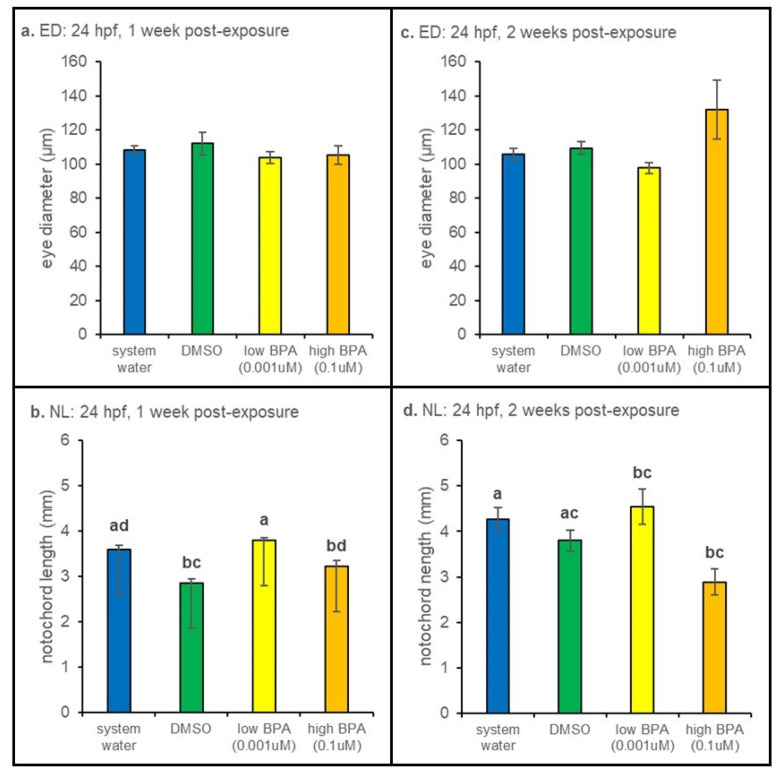
Twenty-four hours postfertilization anatomical measurements. (**a**) Larvae exposed at 24 hpf did not display any differences in eye diameter after 1 week of recovery. (**b**) However, larvae in the low BPA treatment group were significantly larger than vehicle controls and larvae exposed to high BPA (*p* < 0.001) at 1 week post-exposure. (**c**) After 2 weeks, larvae exposed at 24 hpf still displayed no differences in eye diameter, despite the larger values in the high BPA treatment group. (**d**) Larvae exposed to high BPA had the smallest mean length at 2 weeks post-exposure. Significant differences were noted across treatments (*p* = 0.006), however these values were not different from the vehicle control. At each recovery point n = 5 for all treatment groups. Differences in notochord length and eye diameter across treatment were assessed using a one-way ANOVA followed by a Tukey post hoc test at each exposure age. Eye diameter measurements were normalized to notochord lengths prior to statistical analysis. All values are reported as mean ± SE. Different letters denote significance.

When exposed to high BPA as 24 hpf embryos, larval notochord lengths measured 1 week after removal from treatment were significantly smaller (*p* < 0.001) than measurements of larvae in the low BPA group (Figure 2b). Larvae within the low BPA exposure group were also significantly larger than vehicle controls.

Notochord length was also reduced in larvae embryonically exposed to high BPA and measured after 2 weeks of recovery, though the result was only significantly different from water-treated controls (*p* < 0.001; Figure 2d). No differences in eye diameter were noted at either the 1-week (*p* = 0.642; Figure 2a) or 2-week (*p* = 0.093; Figure 2c) time points.

#### 3.2.2. 72 hpf Exposures

**Figure 3:**
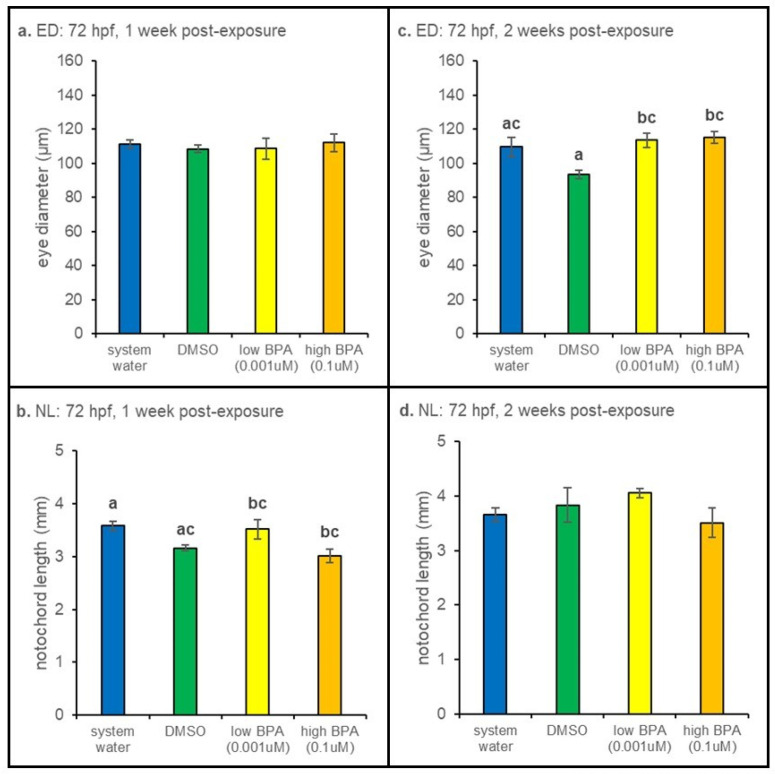
Seventy-two hours postfertilization anatomical measurements. (**a**) Larvae exposed at 72 hpf displayed no significant differences in eye diameter after 1 week of recovery. (**b**) Though significant differences in length were observed (*p* = 0.014), BPA- and vehicle-treated animals were not different. (**c**) Eye diameter was significantly increased in both low and high BPA exposure groups at the 2 week time point (*p* = 0.006). (**d**) No significant differences in notochord length were evident after 2 weeks of recovery. At each recovery point n = 5 for all treatment groups. Differences in notochord length and eye diameter across treatment were assessed using a one-way ANOVA followed by a Tukey post hoc test at each exposure age. Eye diameter measurements were normalized to notochord lengths prior to statistical analysis. All values are reported as mean ± SE. Different letters denote significance.

Measurements of larvae exposed to BPA at 72 hpf revealed a significant (*p* = 0.014) decrease in notochord length after 1 week of recovery compared water-treated controls (Figure 3b). No differences in notochord length were observed at the 2 week postexposure timepoint (Figure 3d). However, at 2 weeks postexposure, eye diameter was significantly larger in both BPA treatment groups (*p* = 0.006; Figure 3c). No differences in eye diameter were noted 1 week postexposure (Figure 3a).

3.2.3. 7 dpf Exposures

**Figure 4:**
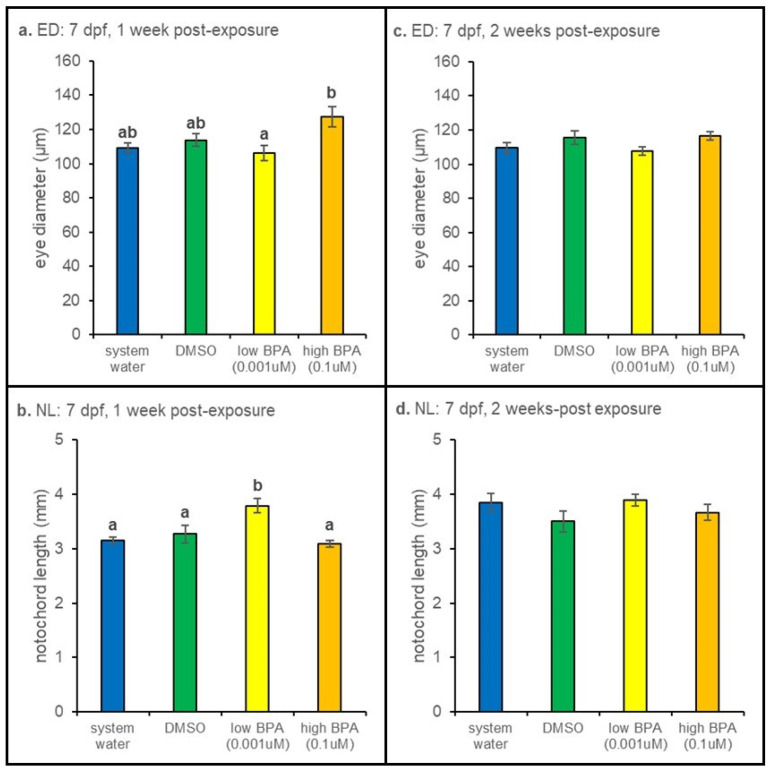
Seven days postfertilization anatomical measurements. (**a**) Compared to controls, eye diameter was not increased in either BPA group after 1 week of recovery, though the high BPA exposure group had significantly larger eyes than the low BPA exposed larvae (*p* = 0.022). (**b**) After 1 week of recovery, notochord length was significantly increased larvae exposed to low BPA (*p* < 0.001). No significant differences were noted after 2 weeks of recovery for either (**c**) eye diameter or (**d**) notochord length. At each recovery point n = 5 for all treatment groups except for DMSO, in which n = 4. Differences in notochord length and eye diameter across treatment were assessed using a one-way ANOVA followed by a Tukey post hoc test at each exposure age. Eye diameter measurements were normalized to notochord lengths prior to statistical analysis. All values are reported as mean ± SE. Different letters denote significance.

When BPA exposure began at 7 dpf, eye diameter was increased in the high BPA group at 1 week post-exposure compared to measurements from low BPA treated larvae (*p* = 0.022; Figure 4a). However, at this time point, low BPA treated larvae were larger than those in the high BPA group (*p* < 0.01; Figure 4b). Notochord length and eye diameters were not different at the 2 week recovery timepoint (Figure 4c,d). 

### 3.3. Behavioral Responses

#### 3.3.1. 24 hpf Exposures

**Figure 5:**
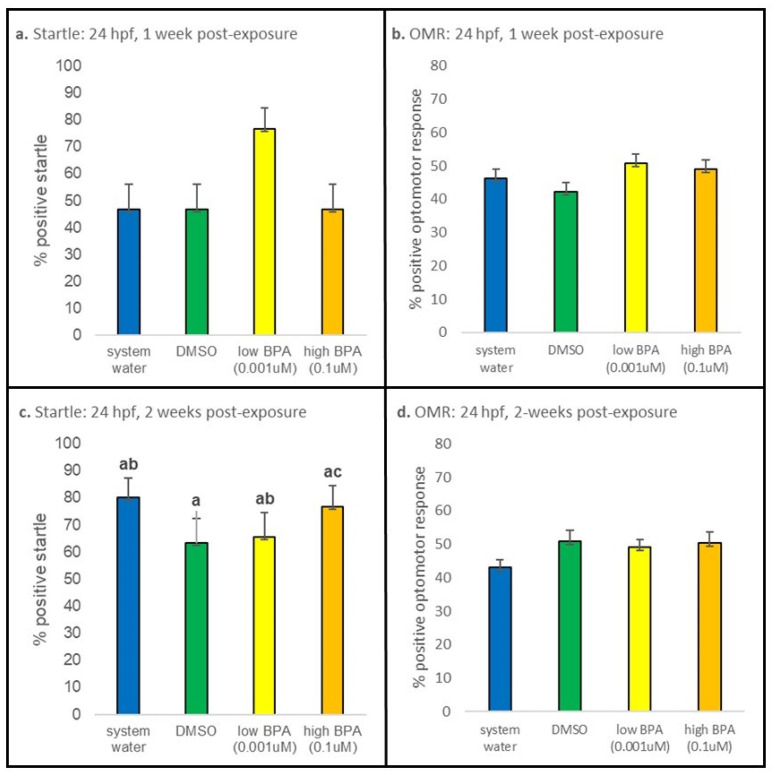
Twenty-four hours postfertilization behavioral results. (**a**) After 1 week of washout, larvae exposed at 24 hpf did not display statistically different startle responses. (**b**) No differences were noted in OMRs displayed by after 1 week of recovery. (**c**) At 2 weeks post-exposure, larvae exposed to high BPA displayed increased startle responses compared to larvae exposed to low BPA only (*p* = 0.001). (**d**) No differences in OMRs were observed in larvae exposed at 24 hpf at the 2 week time point. At each recovery point n = 30 for all treatment groups. Startle responses were analyzed using a one-way ANOVA performed separately for each age and treatment group; if significance was noted, a Tukey post hoc test was performed to identify differences. OMR was analyzed using a one-way ANOVA, followed by a post hoc multiple comparison (with a Bonferroni correction). All values are reported as mean ± SE. Different letters denote significance.

After 1 week of recovery, larvae that were exposed at 24 hpf displayed no differences in startle responses or OMRs (Figure 5a,b). The percentage of larvae displaying a positive OMR was also not different at 2 weeks postexposure (Figure 5d). However, startle responses between BPA treatment groups were found to be significantly different at the 2 week time point (*p* = 0.001; Figure 5c), though these values were not significantly different from controls. 

#### 3.3.2. 72 hpf Exposures

**Figure 6:**
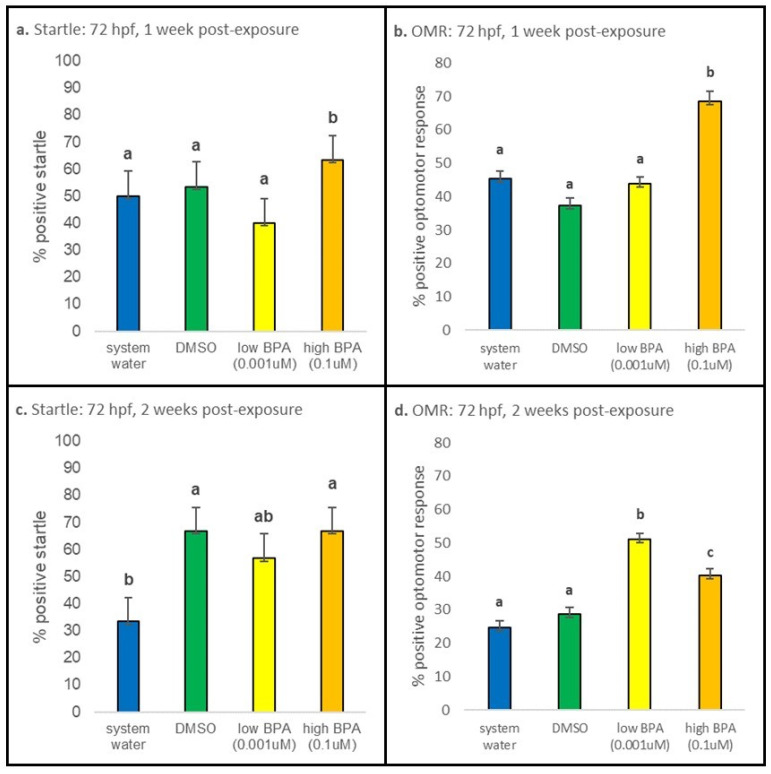
Seventy-two hours postfertilization behavioral results. (**a**) After 1 week of recovery, larvae exposed to the high concentration of BPA at 72 hpf displayed statistically increased startle responses relative to all other treatment groups (*p* < 0.001). (**b**) There was also a significant increase in positive OMRs (*p* < 0.001) in the high BPA group. (**c**) At the 2 week recovery time, larvae exposed to high BPA displayed significantly increased startle responses compared to low BPA exposed larvae (*p* = 0.003), though neither response was different from the vehicle control. (**d**) The percentage of larvae displaying a positive OMR was significantly increased in both the low BPA (*p* < 0.001) and high BPA (*p* = 0.001) treatment groups, with the greatest response observed in larvae exposed to low BPA. At each recovery point n = 30 for all treatment groups. Startle responses were analyzed using a one-way ANOVA performed separately for each age and treatment group; if significance was noted, a Tukey post hoc test was performed to identify differences. OMR was analyzed using a one-way ANOVA, followed by a post hoc multiple comparison (with a Bonferroni correction). All values are reported as mean ± SE. Different letters denote significance.

At 1 week postexposure, larvae exposed at 72 hpf displayed statistically increased startle responses in the high BPA treatment group compared to all other treatment groups (*p* < 0.001; Figure 6a); differences were not noted between the controls and low BPA group. At 2 weeks postexposure, startle responses were significantly reduced in water-treated vs. vehicle-treated controls and between low BPA vs. high BPA treatment groups (*p* = 0.003; Figure 6c). At 1 week postexposure, the percentage of positive OMRs was significantly elevated in the high BPA group (*p* < 0.001; Figure 6b). At 2 weeks postexposure, there is still a significantly greater number of high BPA exposed larvae displaying positive OMR (*p* = 0.001; Figure 6d). However, there is now also a significant increase in positive OMR displayed by low BPA exposed fish (*p* < 0.001; Figure 6d). The percentage of fish displaying a positive OMR is also significantly different between low and high BPA groups at 2 weeks postexposure (*p* = 0.001; Figure 6d).

#### 3.3.3. 7 dpf Exposures

**Figure 7:**
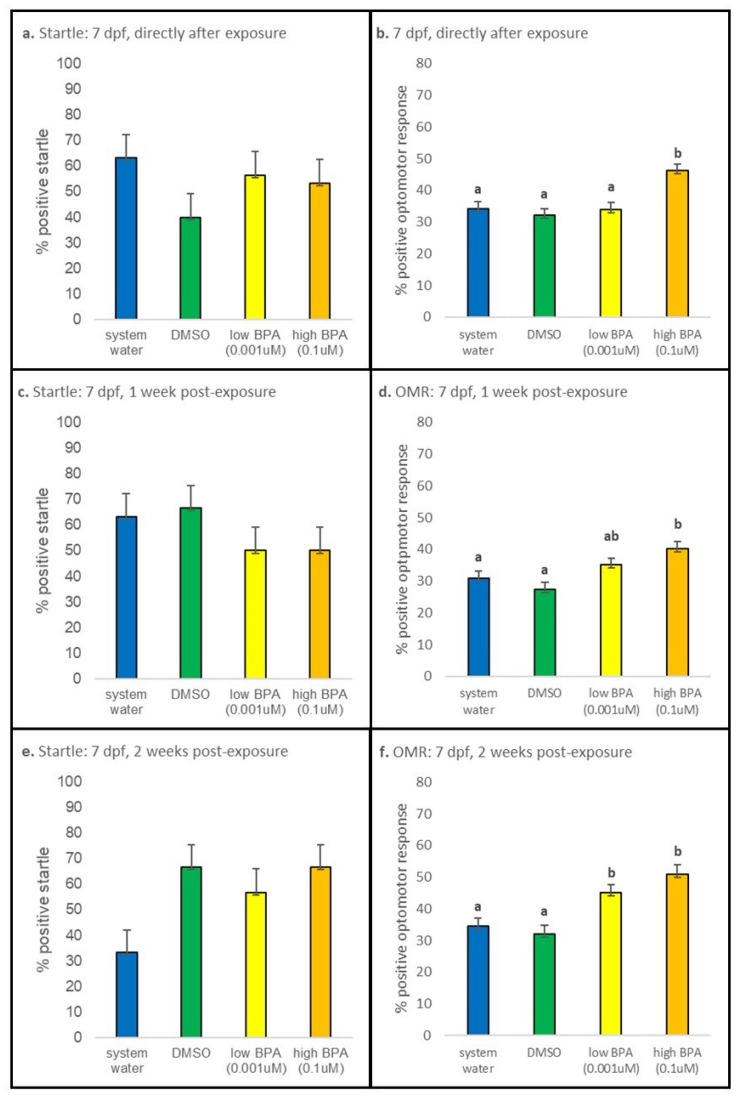
Seven days postfertilization behavioral results. No differences in startle responses were observed in larvae assessed **a.** immediately, **c.** after 1 week of recovery, or **e.** after 2 weeks of recovery when exposure occurred at 7 dpf. In contrast, **b.** larvae exposed to high BPA displayed a significant increase in the percentage of fish displaying a positive OMR when assessed immediately after exposure (*p* < 0.001). Increased numbers of larvae displaying a positive OMR were also observed **d.** after 1 week of recovery (*p* = 0.020) and **f.** after 2 weeks of recovery in larvae exposed to both low (*p* < 0.036) and high (*p* < 0.001) BPA. At each recovery point n = 30 for all treatment groups. Startle responses were analyzed using a one-way ANOVA performed separately for each age and treatment group; if significance was noted, a Tukey post hoc test was performed to identify differences. OMR was analyzed using a one-way ANOVA, followed by a post hoc multiple comparison (with a Bonferroni correction). All values are reported as mean ± SE. Different letters denote significance.

Startle responses were not significantly different across all treatment groups in larvae exposed at 7 dpf and tested either immediately after exposure or after 1 week and 2 weeks of recovery (Figure 7a,c,e). Immediately after exposure, however, larvae exposed to high BPA displayed a statistically significant increase in positive OMRs (*p* < 0.001; Figure 7b). A similar result was observed after 1 week of recovery (*p* = 0.020; Figure 7d). After 2 weeks of recovery, low and high BPA exposed larvae displayed statistically increased positive OMRs compared to control groups (*p* < 0.036 and *p* < 0.001, respectively; Figure 7f), though the difference between the low and high BPA groups was not significant (*p* = 0.882).

## 4. Discussion

Overall, our data suggest age- and concentration-dependent effects of developmental BPA exposure on the visual system. When exposure occurred at 72 hpf and 7 dpf, initial increases in optomotor responses were observed 1 week postexposure in larvae exposed to high (0.1 µM) BPA (Figure 6b and Figure 7b); after 2 weeks, a difference in OMRs in larvae treated with low (0.001 µM) BPA was also observed (Figure 6b and Figure 7b), identifying a slow-to-develop effect. These changes were correlated with changes in eye diameter (Figure 3c and Figure 4a). In contrast, embryonic (24 hpf) exposure affected overall growth of larvae (Figure 2b,d) but did not alter optomotor responses (Figure 5b,d). Taken together, these results suggest that transient, developmental exposure to sublethal, environmentally relevant concentrations of BPA have significant effects on larval zebrafish morphology and visually guided behavior. 

BPA is a well-known environmental contaminant and one of the highest volume chemicals produced [5,35]. Measurable levels are found in soil, water, and atmospheric samples worldwide [67]. Exposure to humans and animals is significant [5] and early exposure leads to developmental [5,35] and transgenerational [41] effects. Concentrations reported in aquatic environments range from µg/L [6] to ng/L [67] and most studies examining biological effects of BPA use µM concentrations. 

Published experiments with zebrafish report BPA is rapidly taken up when exposure occurs through tank water. Detectable levels are evident after 2 h and steady-state levels occur after 24 h of exposure [68]. Acute developmental exposure alters zebrafish lateral line [52] and otolith [50] development and exposure during the first 24 hpf affects development of the midbrain, otic vesicles, and somites [49]. Transient developmental exposure, within the hours/days postfertilization, also has long-term effects on locomotor activity [46,47,48,58], hair cell development [52], and brain development [49,58]. Other long-term consequences include altered inflammation [69] and reproductive effects [70,71]. We found no differences in survival across treatment groups when exposure occurred at 24 hpf. However, daily survival varied widely across treatment groups when exposure occurred at 72 hpf or 7 dpf. In larvae exposed at 72 hpf, those exposed to 0.001 µM (low) BPA group displayed the sharpest decrease in survival starting 1 week post-exposure, which resulted in overall treatment differences. This suggests concentration-dependent differences, which agrees with the experimental endpoints measured (see below). In larvae exposed at 7 dpf, those in the vehicle (0.0003% DMSO) group displayed the sharpest changes in daily survival, though overall survival at 1 week and 2 weeks post-exposure was not significantly different. DMSO (0.00075%) used as the vehicle in a study of estrogenic signaling in juvenile salmon was found to change expression of estrogenic markers [72], suggesting direct effects. However, we found that larvae in the vehicle control group had responses that were not significantly different from water controls for all measurements, with two exceptions. While we cannot completely discount a potential direct effect of DMSO, examination of the data overall suggests DMSO may not be having an individual effect in our experiments. 

We have identified an effect of transient developmental exposure to BPA on the visual system. These effects were localized to specific exposure ages: 72 hpf and 7 dpf. Zebrafish larvae hatch at 72 hpf, an age where when all retinal cell types are present [24] and retinal ganglion cell axons have innervated the optic tectum [25] suggesting functional circuitry. Visually guided optomotor responses can be recorded at 7 dpf [55], an age when zebrafish larvae have exhausted their yolk sac and are actively feeding. Estrogen circuitry, the target of BPA, is also functional at these ages. Estrogen is synthesized by the aromatization of testosterone by the enzyme aromatase (estrogen synthase). Zebrafish brain expresses high levels of aromatase [73,74,75] and this expression occurs throughout the life of the fish [76]. Aromatase expression begins at 24-48 hpf, when estrogen receptor mRNA expression also begins [77,78,79,80]; expression of GPER (G-protein coupled estrogen receptor) occurs at 72 hpf [81]. Aromatase protein can be detected in retina at 7 dpf using immunocytochemistry [56]. BPA, as an estrogen agonist, is able to bind all estrogen receptors [21,44,82,83], activating intracellular signaling and increasing expression of both aromatase and estrogen receptors [21,45,57,58,84,85]. In this way, BPA increases estrogen signaling. The specific effects of BPA at 72 hpf and 7 dpf suggests estrogen receptors are functional at these ages, that BPA binds to these receptors, and that the effect of BPA is prolonged, lasting up to 2 weeks after removal from treatment. 

Embryonic exposure (24 hpf) affected larval growth. Low dose (0.001 μM) BPA resulted in significantly larger larvae when measured 1 week and 2 weeks postexposure whereas, exposure to high (0.1 μM) BPA had the opposite effect, resulting in significantly smaller larvae. These results agree with another study examining embryonic BPA exposure, which identified shorter larvae and wider somites in larvae treated within the first 24 hpf [49]. However, we found no differences in eye diameter or optomotor responses. While this could be due to an inability of BPA to cross the chorion, as reported for butachlor, another endocrine disruptor [86], differences in growth measurements suggest this is not the case. Rather, the timing of estrogen receptor expression, which is beginning this exposure age, suggests these receptors may not be functional. In agreement with this, we previously showed that transient exposure to the aromatase inhibitor formestane (4-OH-A) at 24 hpf did not alter optomotor responses in adult zebrafish [87], consistent with an absence of functional estrogen synthesis and signaling at this exposure age.

Differences in growth were also observed when exposure occurred at 72 hpf and 7 dpf. Larvae exposed to high (0.1 µM) BPA at 72 hpf were smaller after 1 week of recovery. Eye diameter measurements, in contrast, revealed a slow-to-develop effect with increased eye measurements observed 2 weeks after removal from treatment. Larvae exposed at 7 dpf revealed transient morphological effects, though now differences in eye diameter noted at 1 week postexposure were absent at 2 weeks postexposure (Figure 4a,c). Overall, we observed a non-monotonic effect [46,47] on growth, where exposure to high BPA caused a decrease in notochord length, while exposure to low BPA caused an increase in notochord length, when measured 1 week post-exposure. 

These results suggest concentration-dependent effects of BPA, consistent with published reports [9,69]. We similarly observed concentration-dependent effects of BPA exposure in our OMR data, in addition to a time-dependent component. We observed an early, and consistent, increase in the number of larvae displaying a positive OMR with high dose BPA but delayed effects of low dose BPA that were not observed until 2 weeks after treatment. At this time, the number of larvae displaying a positive OMR was also increased. Published work reports 0.1 µM BPA induces hyperactivity in zebrafish larvae when exposure occurs before 36 hpf [58] or before 58 hpf [46], suggesting that the observed increase in OMR responses may be due to an overall increase in activity. To assess this, we examined startle responses in exposed larvae. We found BPA-induced increases in startle responses in two groups: (1) larvae exposed at 72 hpf and tested 1 week later and (2) larvae exposed at 24 hpf and tested 2 weeks later. Since larvae in the first group also showed increased OMR, it is possible that BPA-induced hyperactivity in this treatment group contributed to this response. However, larvae exposed to either high (0.1 µM) or low (0.001 µM) BPA at 72 hpf and tested 2 weeks later displayed significant differences in OMR only, as did all larval groups exposed at 7 dpf. We interpret these results as an effect of BPA on OMR circuitry, suggesting a specific effect on vision-based behaviors. 

Our data revealed a clear persistent effect of BPA exposure. However, the absence of any immediate effects of the low concentration of BPA was unexpected. We do not know the specific mechanism underlying the delayed effect, there are two possibilities. First, there could be differential activation of estrogen receptors (ER) by BPA. Though BPA binds to all ERs, including GPER [88], BPA binds ERα as an agonist, but binds ERβ as an antagonist [89] and these two receptor types participate in estrogen dependent signaling through distinct molecular circuits [90]. Zebrafish ERs (zfERα, zfERβ1, and zfERβ2) have different affinities for estradiol [91], suggesting there may also be differences in affinity for BPA. ER activation results in altered gene expression; whereas GPER activation is associated with more immediate effects in cells, such as altered calcium signaling [88,92]. In addition, BPA strongly binds to estrogen related receptors (ERRγ) [93] and activation of ERRβγ affects behavior in both larval [46] and adult [47] zebrafish. Differences in BPA binding have been observed when exposure occurs within with nmol/L to mmol/L range [94], consistent with concentrations used here. Thus, is it possible that BPA differentially bound to all functional ER and ERR types in zebrafish larvae, activating different pathways, which may have resulted in the time-dependent effects observed here. Second, BPA may have cross-reacted with other receptor types, such as thyroid hormone and androgen receptors [95,96]. While we do not anticipate a role of thyroid receptors, as BPA binds these receptors at doses much higher than those used here (10 µM [35,97,98]), androgen receptor activation is a possibility. Low doses of BPA increases androgen receptor expression [34]. Kinch et al. [58], working in zebrafish, found that 0.0068 µM BPA was able to agonize androgen receptors, and increase aromatase (cyp19a1b) expression, when exposure during specific time periods before 36 hpf. The overlaps in exposure age and BPA concentration with our study is worth mentioning and suggests cross-reactivity may be occurring at the doses used in our preparation. We are currently performing molecular analyses of exposed tissues to identify the intracellular mechanisms underlying the observed behavioral responses.

## 5. Conclusions

Overall, our results indicate that transient, developmental exposure to sublethal and environmentally relevant concentrations of BPA alters larval morphology and visually guided behaviors. Embryonic exposure reduced growth, while exposure immediately after hatching (72 hpf) and during the early larval stage (7 dpf) were found to target visually guided circuitry with concentration-dependent effects. At these ages, BPA exposure increased the number of larvae displaying a positive OMR and increased eye diameter measurements. Overall, these data suggest functional changes at one or more levels of visual development, sensation, or processing. Though significantly deleterious effects were not noted, the time- and concentration-dependent effects noted may support the dual function of BPA as both an estrogenic agonist and antagonist.

## Data Availability

The data presented in this study are available upon request from the corresponding author.

## Abbreviations

4-OH-A	4-hydroxyandrostenedione
BPA	bisphenol-A
dpf	days post-fertilization
DMSO	dimethyl sulfoxide
EDCs	endocrine disrupting compounds
ER	estrogen receptor
ERRγ	estrogen related receptors
ED	eye diameter
GPER	G-protein coupled estrogen receptor
hpf	hours post-fertilization
NL	notochord length
OMR	optomotor response
